# The Development of a Forceps-Adaptable Pressure Device for Instrumental Delivery: A Proof-of-Concept Study for Clinical and Educational Applications

**DOI:** 10.3390/s24237839

**Published:** 2024-12-08

**Authors:** Mathieu Hivert, Cyril Bengler, Julien De Jonckheere, Franck Gaultier, Marie Pécout, Olivier Mayeur, Chrystèle Rubod

**Affiliations:** 1 Jeanne de Flandre Hospital, Faculté de Médecine, University of Lille, Avenue Eugène Avinée, 59000 Lille, Francemarie.pecout@chu-lille.fr (M.P.);; 2 Département de Médecine, Université de Lille, 42 Rue Paul Duez, 59000 Lille, France; 3 CIC-IT Lille, Boulevard du Professeur Jules Leclercq—CS 70001, CEDEX, 59037 Lille, France; 4 CITC Lille, 172 Avenue de Bretagne, 59000 Lille, France; 5 Laboratoire de Mécanique Multiphysique Multiechelle (LaMcube), UMR 9013, Centrale Lille, CNRS, Université de Lille, 59000 Lille, France

**Keywords:** obstetric instrumentation, fetal head pressure, clinical practice, maternal and neonatal outcomes

## Abstract

Objective: To develop and validate a device that measures the pressure exerted by forceps on the fetal head for clinical use. Background: The lack of clinical tools to quantify forceps pressure on the fetal head may impact maternal and neonatal outcomes. Existing studies have not measured the direct contact pressure between forceps blades and the fetal head, highlighting the need for innovation. Methods: We integrated fluid pressure transducers into obstetric forceps using fluid-filled tubing encased in flexible silicone socks attached to the blades. Tubing materials—polyvinyl chloride (PVC) and polyurethane (PU)—and fluids (air and water) were tested with both biocompatible and non-biocompatible silicone socks. An onboard electronic board collected pressure data and transmitted them via Bluetooth for real-time analysis. The system was evaluated on a custom-built bench simulating forceps application. Results: Air-filled tubing exhibited significant drift and low accuracy due to air compressibility. Water-filled PU tubing reduced drift but was still suboptimal. Water-filled PVC tubing with both types of silicone socks provided the best results, showing minimal drift and a strong correlation between measured pressures and applied forces. Conclusions: The developed device represents a significant advancement, as no existing system measures the pressure exerted by forceps blades on the fetal head. By effectively measuring pressure across the entire contact surface in real time, it offers applications in both training and clinical practice. The device allows for objective feedback, potentially improving the safety and efficacy of forceps deliveries. Future work includes comprehensive mannequin tests and eventual in vivo studies to validate its effectiveness in realistic settings, aiming to enhance obstetric training and reduce maternal and neonatal complications.

## 1. Introduction

The use of forceps for fetal delivery varies according to team practices. These differences can be observed internationally, nationally, and sometimes within the same department. There are no official European or global statistics on their use. Forceps rates can be found in recent national perinatal reports from several countries. In 2016, the proportion of forceps deliveries was 3.36% in France [1], 3.92% in Spain [2], 3.5% in Ireland, 7.3% in the United Kingdom [3], and 0.5% in the United States [4]. Additionally, the rate of cesarean births is increasing worldwide [5]. Forceps delivery is associated with an increased risk of severe perineal tears and long-term pelvic static disorder in the mother as well as rare but potentially serious neonatal complications [6,7,8,9,10,11,12,13,14,15]. If the technique is correct and the indications and contraindications are respected, scientific societies agree that forceps extraction is a safe and recommended procedure that reduces the number of emergency cesarean sections [16,17,18].

Training in forceps delivery is essential for their safe use. However, to our knowledge, there is currently no clinical tool available to objectively assess the quality of forceps delivery in the labor room. This scarcity in the literature confirms the urgent need for new research and validates the importance of our work in filling this scientific gap. Forceps delivery is usually an emergency procedure, and the time required to complete it should be as short as possible. In this context, it is difficult to entrust forceps delivery to an inexperienced OB/GYN intern, especially as it is currently impossible for the senior physician supervising the procedure to provide objective feedback on the force or axis of traction applied during instrumental delivery. A study published in 2015 described the practices of 12,728 OB/GYN residents in the USA who graduated between 2002 and 2012. It showed a significant decrease in vaginal deliveries (−17%) and instrumental deliveries with forceps (−64.7%) and vacuum (−26%) between 2002 and 2012 [19]. Despite the rapid development of simulation-based training on sophisticated and sometimes instrumented mannequins, there is currently no solution that allows for the objective assessment of student performance during forceps delivery practice.

The aim of our work is to develop a device to measure the pressure exerted by forceps on the fetal head, first in a teaching environment and then, after validation, in current clinical practice. Therefore, we developed, tested, integrated, and validated the pressure measurement system on a test bench.

## 2. Materials and Methods

### 2.1. Sensor Selection, Testing, Validation, and Integration

The selection of the sensors to be used and their placement on the forceps was based on the specifications initially developed by the medical team. The main data we wanted to collect were on the pressure exerted on the fetal head by each blade of the forceps during the procedure. In fact, some fetal complications are related to excessive pressure or the asymmetrical positioning of the blades, resulting in an unequal distribution of the compression forces applied to each side of the head. The sensors were selected accordingly. After selecting the appropriate sensors, we first tested them in a non-configured mode to verify that they could meet our expectations and in order to understand the various external factors influencing the measurements obtained.

After this initial test phase, we adapted these sensors to the forceps; we had to check that the final ergonomics were acceptable before validating our choice of sensors. We then performed parameterized tests on a test bench. The goal was to validate the sensors in terms of measurement reliability and repeatability. From the beginning, the device was designed for in vivo use; all equipment had to be sterile or sterilizable, and parts that came into contact with the mother and newborn had to be made of biocompatible materials. The device also had to be quick and easy to use, as the use of forceps is often an emergency procedure. This constraint guided our ergonomic choices during development. This study is preliminary and primarily focused on the development and initial validation of the device prototype.

#### 2.1.1. Pressure-Measuring Equipment

**Figure 1:**
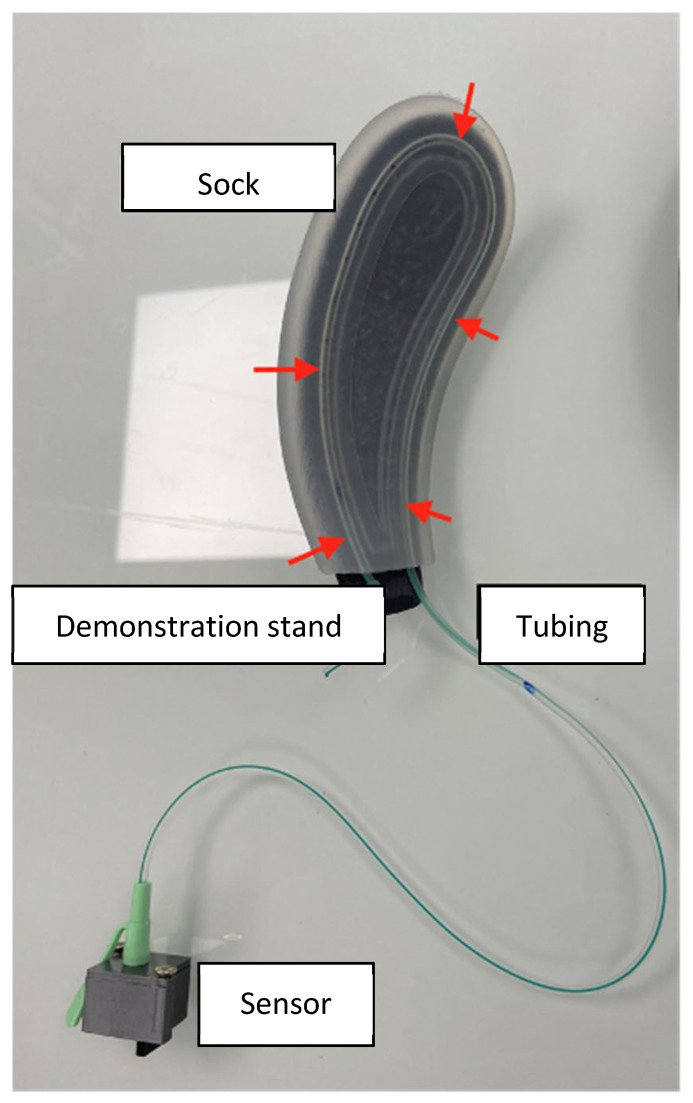
Tubing and support sock on a demonstration stand and the sensor integrated into an adapted box. The red arrows indicate the path of the tubing in the sock.

Pressure on the fetal head is not evenly distributed over the surface of the forceps blade and is not systematically equal between the two branches. We therefore decided to measure the pressure applied to the entire surface of the forceps blade using fluid pressure transducers (air or water). Three elements were required for this (Figure 1):

- A tubing chamber, filled with a fluid and sealed at its distal end.
- A pressure sensor connected to the proximal part of the tube. This sensor measures the pressure directly inside the tube and is housed in a box that allows it to be connected to an electronic circuit board.
- An attachment system designed as a flexible silicone sock, accommodating the tubing and stabilizing it over the entire surface of the blade in contact with the fetal head.

#### 2.1.2. Data Reception and Retransmission

Once the pressure measurement system had been defined, the data needed to be collected and exported to an analysis tool. The pressure sensors did not have an autonomous communication system. Therefore, a battery-powered electronic board was selected and programmed to receive data from the pressure sensors in real time and transmit them to a computer. To miniaturize the forceps and improve ergonomics, the electronic board had to be very small. It contained only the essential functions.

#### 2.1.3. Data Analysis

Raw data sent via Bluetooth Low Energy by the electronic card on the forceps were collected, recorded, and analyzed in real time by a computer. Data extraction software was developed for graphical display.

#### 2.1.4. Data Representation

The results of the analysis were then presented to the physician in a synthetic and intuitive manner so that they could be integrated into the management process.

### 2.2. Selection of Pressure Capture System Components

#### 2.2.1. Tubing

The most suitable tubing for our project was the Levin pediatric gastro-duodenal tube, first because it is available in many sizes, diameters, and materials and second because it is biocompatible.

The diameter of the tubing had to be fine enough to fit over the forceps without increasing their thickness but wide enough to prevent the lumen of the tubing from collapsing at low pressures. We therefore opted for a diameter of 6 Fr (i.e., 2 mm in diameter). The tubing selected was initially 125 cm long. As the elasticity and plasticity characteristics of tubing in different materials cannot be predicted, we tested two available materials: polyvinyl chloride (PVC) (VYGON part no. 391.06) and polyurethane (PU) (VYGON part no. 1362.067). The tubing had to be rigid enough not to collapse under pressure but elastic enough to accept the deformations caused by repeated pressure, while at the same time recovering its initial shape to keep hysteresis to a minimum.

#### 2.2.2. Pressure Sensors

For ergonomic reasons, we chose Honeywell’s model SSCDANV015PGSA3 blood pressure sensor (Honeywell, Charlotte, NC, USA) for all of our tests. The range of pressures measured was from 0 to 15 PSI (104 kPa), compatible with the few estimates available in the literature for this application. This sensor is already used in the medical industry and offers accuracy to within 2%. Initial tests with the miniaturized sensor revealed a ‘water column’ effect due to the length of the tubing. It was possible to correct this effect by placing the sensors directly on the forceps, to limit this differential. From an ergonomic point of view, the presence of long tubes that need to be connected to a remote electronic board could hamper the procedure, so we decided to place the electronic board and pressure sensors directly on the locking system at the forceps joint.

#### 2.2.3. Sock Integration for the Tubing on the Forceps

**Figure 2:**
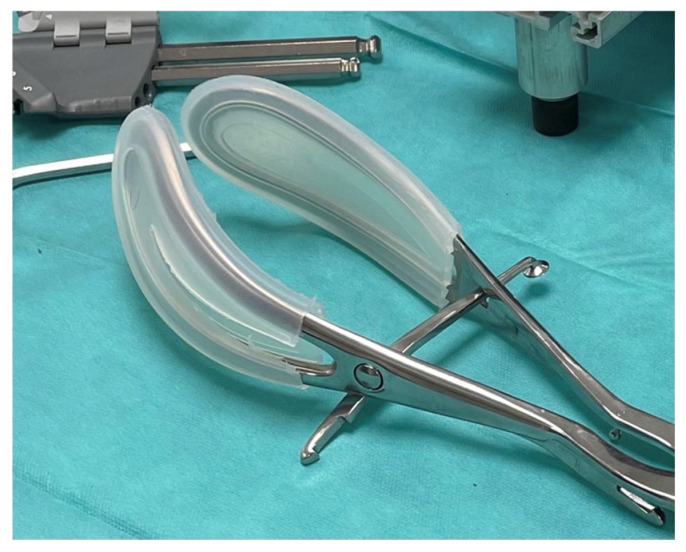
Silicone socks on forceps blades.

The tubing system had to fit over the forceps blades without interfering with instrumental delivery. We developed a system of grooved silicone ‘socks’ (see Figure 2) that fit directly onto the forceps blades. This allowed the tubing to pass over the entire surface that would be in contact with the fetal head. The device was produced using Computer-Aided Design (CAD) tools and 3D printing techniques to create molds for casting various silicones.

Silicone was chosen as the material to integrate the tubing due to its flexibility, which allows for easy adaptation to the shape, and its strength, which holds the tubing on the forceps and its contact zone securely. It can easily be cast in molds of varying sizes, and some types are biocompatible. We tested several non-biocompatible silicones on the first prototypes, including DragonSkin FX Pro, DragonSkin 10 Fast, DragonSkin 30, and DragonSkin 10 NV from the Smooth-On brand (Smooth-On Inc., Macungie, PA, USA). We selected the silicone that produced socks thin enough to avoid excessive pressure absorption. Tests were conducted to optimize the prototype’s ergonomics, allowing the ‘socks’ to be slipped over the forceps easily. Additionally, tests were performed to evaluate the silicone’s tear resistance during instrumental delivery. Among biocompatible silicones, only Biesterfeld’s SkinSil M10 SC met our expectations. Two types were selected for our purpose. One of them is biocompatible and safe for clinical use (SkinSil M10 SC from Biesterfeld), but it is too expensive for teaching purposes. The other reference (10 NV from DragonSkin) is not biocompatible, but its low cost makes it suitable for teaching through simulation. Both silicones adapted well to the forceps. They were strong enough to perform several forceps deliveries in mannequin trials.

#### 2.2.4. Forceps’ On-Board Electronic Card

The chosen electronic board comes with two adapters for connecting the pressure sensors. The data from these sensors are transmitted via a Bluetooth Low Energy connection at a frequency of 10 Hz to the computer that carries out the data analysis.

#### 2.2.5. Integration of the Electronic Card onto the Forceps

**Figure 3:**
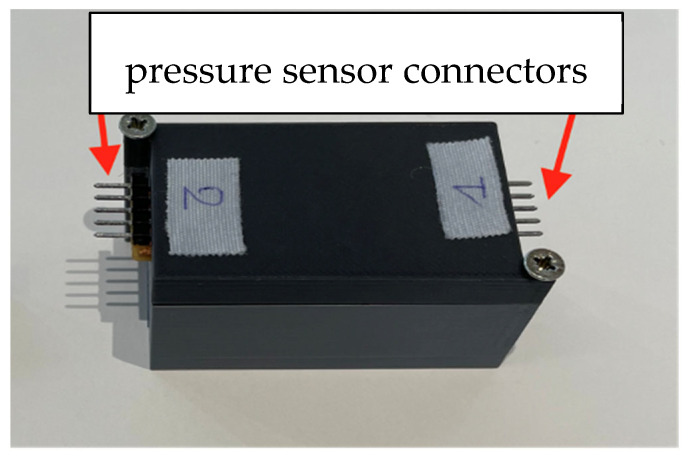
Housing containing the circuit board and battery, with two connectors for pressure sensors.

The pressure transducers and electronic board should be placed directly on the locking system at the forceps joint to minimize the ‘water column’ issue. This made the ergonomics of the setup acceptable. A case was 3D-printed to match the dimensions of the card. This accommodated the electronic board and battery without allowing those elements to move inside. It was fitted with two side openings for connecting the pressure sensors to dedicated connectors on the circuit board (Figure 3). We installed the device at the clamping screw of the forceps arms.

#### 2.2.6. Data Analysis and Representation Software for the User

Data analysis software was developed in the form of a WEB interface developed in HTML and integrating Java scripts and enabled data from each of the pressure sensors to be recorded. Every time the forceps were used, all data were exported and saved. The pressures applied to the blades over time could be determined. The data were also provided to the user in real time.

A color gradient gauge system was developed to measure pressure, ranging from green to red as the pressure increased. Since no reference data were available, the gradients were initially defined arbitrarily. We are currently carrying out tests on a mannequin with a panel of experienced operators to define thresholds. We are working on the web interface to make measuring as simple as possible. We are still in development at this stage.

### 2.3. Pressure Capture System Test Bench

Tests were conducted using tubing plugged at 55 cm, which was the most ergonomic length for integrating and using the device on the forceps. We selected a range of materials for initial testing, including biocompatible silicone. However, this research is still in its early stages, with no immediate plans for clinical use. The purpose of these tests was to compare the reliability, repeatability, and hysteresis of various material combinations.

- Water-filled PVC tubing and a biocompatible silicone sock;
- Water-filled PVC tubing and a non-biocompatible silicone sock;
- Air-filled PVC tubing and a non-biocompatible silicone sock;
- Water-filled PU tubing and a biocompatible silicone sock;
- Water-filled PU tubing and a non-biocompatible silicone sock;
- Air-filled PU tubing and a non-biocompatible silicone sock.

**Figure 4:**
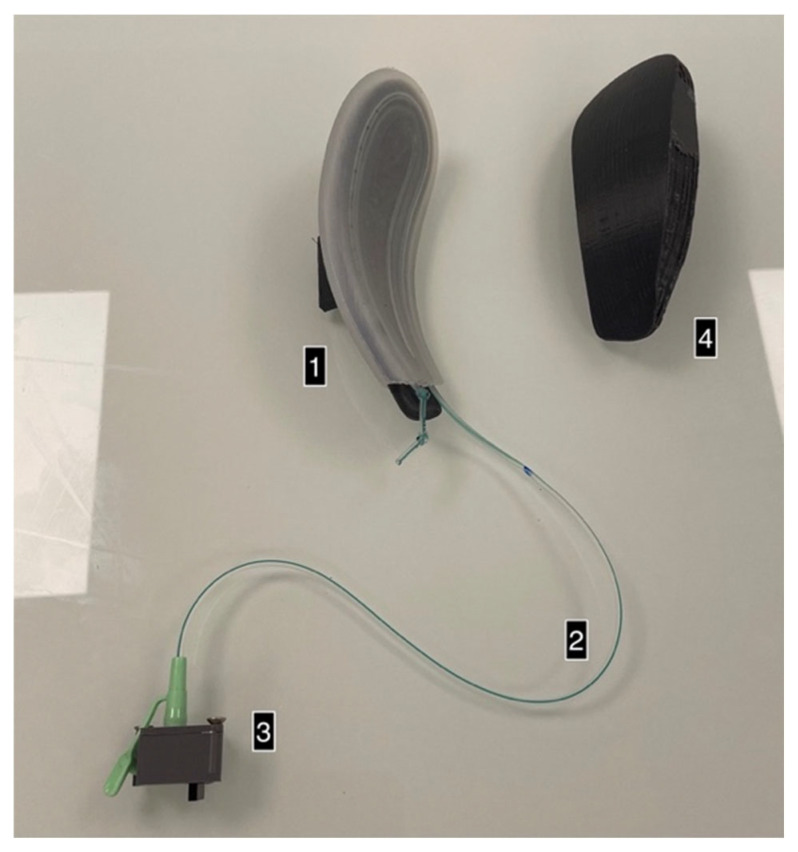
Sock on the support (1), tubing (2), sensor (3), and support surface (4).

**Figure 5:**
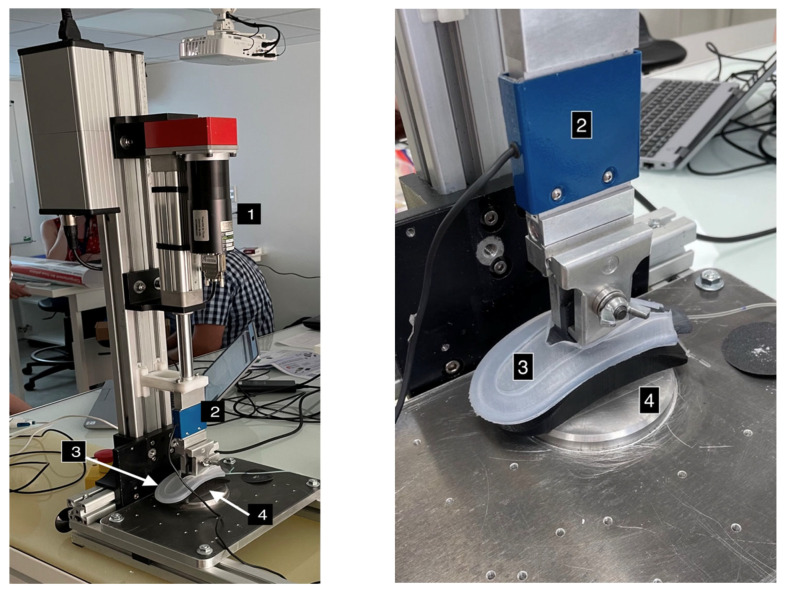
The servomotor (1), force-measuring cell (2), tubing on the support with the sock (3), and contact surface (4).

The tubing was placed in the silicone sock integration system and positioned on a 3D-printed support representing the blades of a set of forceps (Figure 4). This assembly was placed on the moving end of a servomotor-driven cylinder (MAC 140, JVL) fitted with an accurate, calibrated force-measuring cell with a capacity of 100 Newtons (SMT—Type S). A counter-support surface was also 3D-printed, reproducing the contact surface between the forceps and the fetal head (Figure 5).

Cyclic compression cycles were imposed on the device at constant compression and return speeds of 10 mm/min and −10 mm/min. For each displacement distance, we recorded the pressure measured by our sensor in Pascals and the force applied in Newtons using the calibrated sensor installed on the test bench. For each combination of materials and fluids, we carried out five repetitions of five increasing displacement distances. This testing stage enabled us to determine the most reliable combinations to use. For each measurement and each distance repetition, we calculated the maximum and minimum values of the pressure collected by our sensor and the force recorded by the calibrated cell on the test bench. These values were recorded for each compression/decompression cycle.

A Pearson correlation test was then performed. A value of *p* < 0.05 was considered significant. The equation of the correlation line between the pressure transducer and the force transducer was then determined to recalculate the Pascal equivalent of the Newton values provided by the force transducer.

A Bland and Altman test was then performed to estimate the bias (mean error) and the 95% confidence interval. A good correlation between the two measurements was estimated for a bias of less than 1% and 95% confidence interval limits of less than 10%.

In this preliminary study, we conducted Pearson correlation and Bland-Altman analyses across multiple testing conditions and materials (e.g., silicone, PVC) and under different fluid conditions (e.g., water, air) with the aim of assessing measurement accuracy. Given the novel nature of this device and the limited reference data available, we based our sample sizes on the initial validation needs for prototype testing, with the aim of assessing consistency and agreement in controlled settings.

## 3. Results

We compared the pressure data collected by our device with those obtained by the calibrated force transducer. The test results for the various material and fluid combinations are shown below. The orange curves show the force in Newtons measured by the calibrated cell, and the blue curves show the pressure in Pascals measured by our device. For each pair of measurements, we generated a regression curve of the pressure recorded by our sensor as a function of the pressure estimated by the test bench’s force transducer.

### 3.1. Test with Air

**Figure 6:**
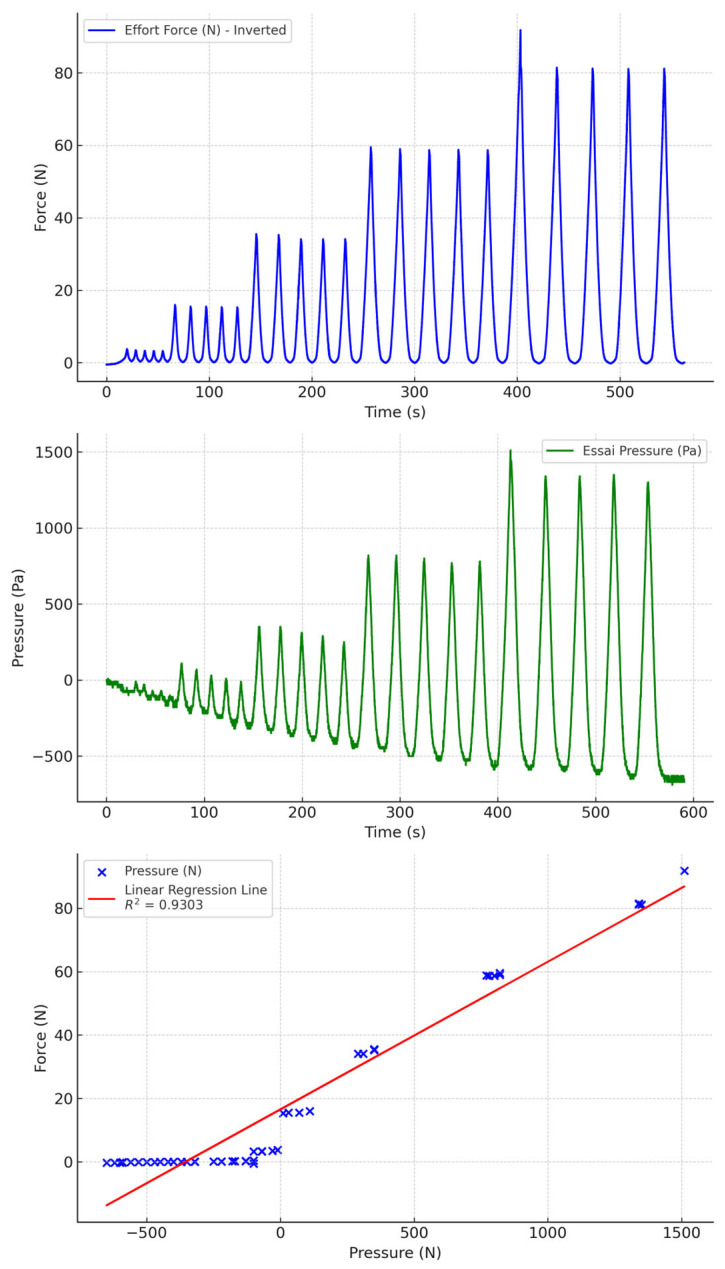
Air-filled PVC tubing with biocompatible socks.

**Figure 7:**
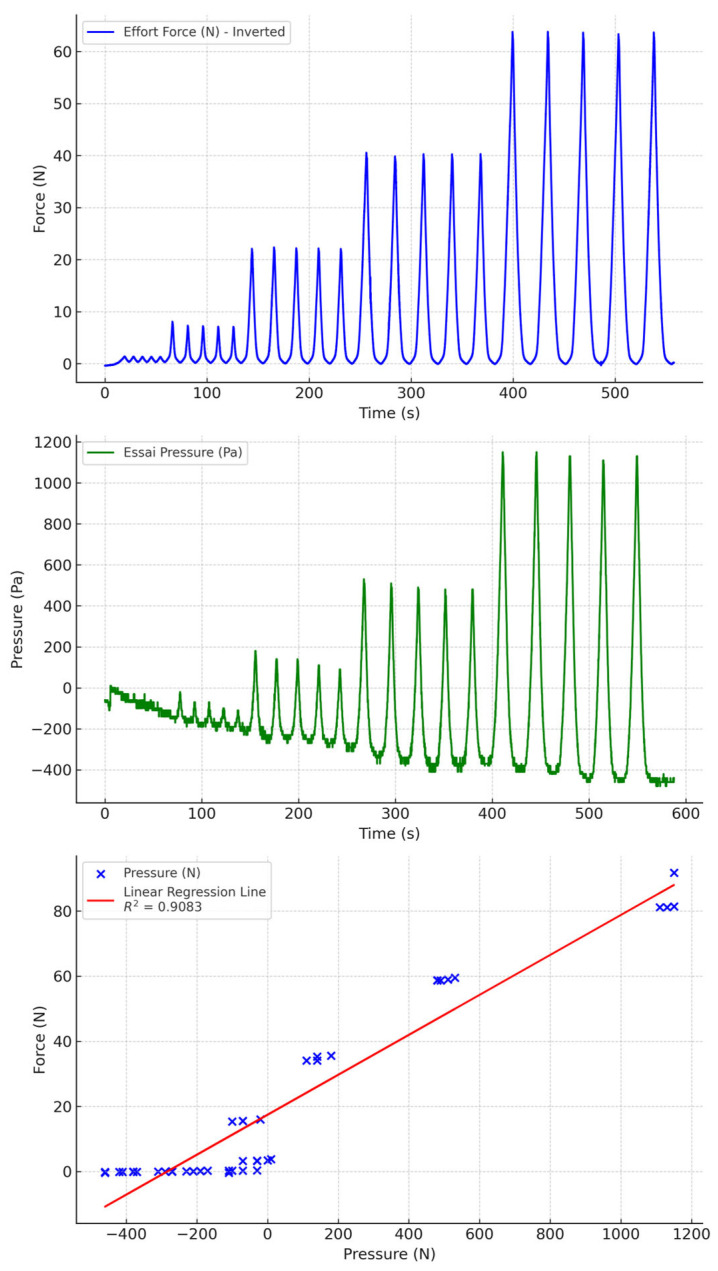
Air-filled PU tubing with biocompatible silicone socks.

When the tubing is filled with air (Figure 6 and Figure 7), there is a considerable drift during compression. Furthermore, the range of pressures measured is low (below 2000 Pa), which can lead to a reduction in measurement accuracy. This is due to the compressibility of air, which partly absorbs pressure, making measurement impossible at low pressures.

### 3.2. Test with Water and PU Tubing

**Figure 8:**
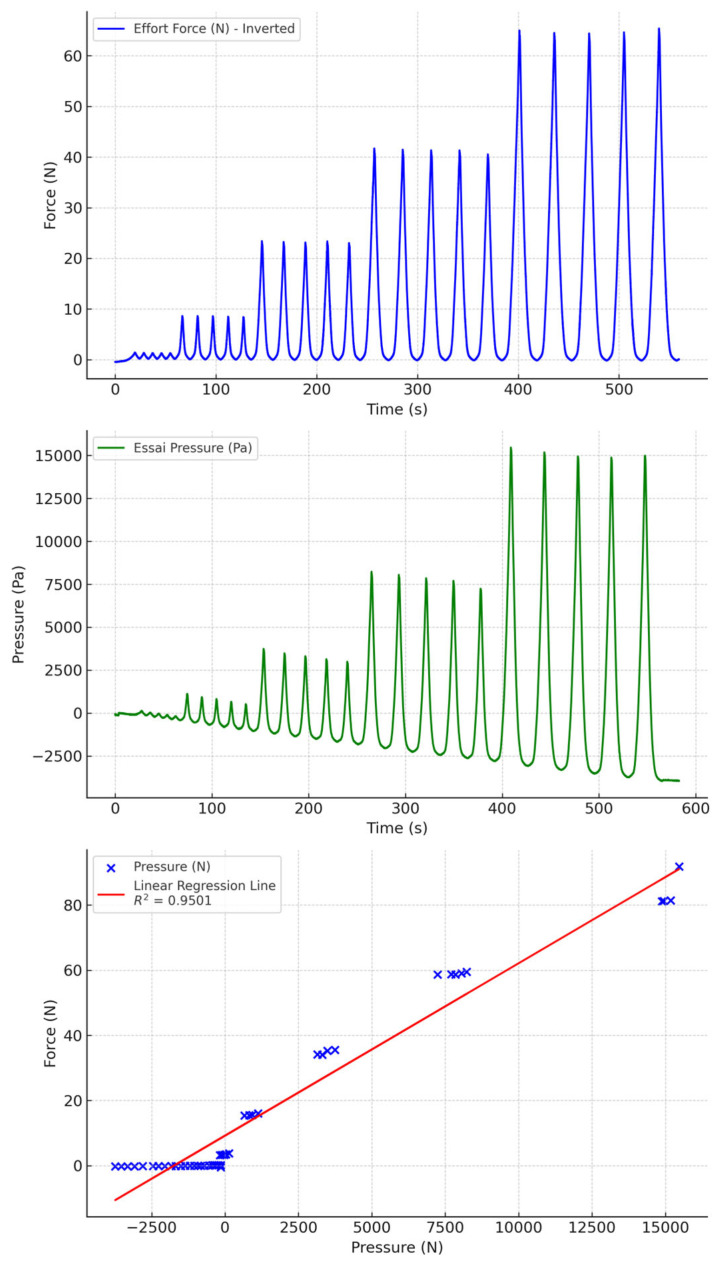
Water-filled PU tubing with biocompatible socks.

**Figure 9:**
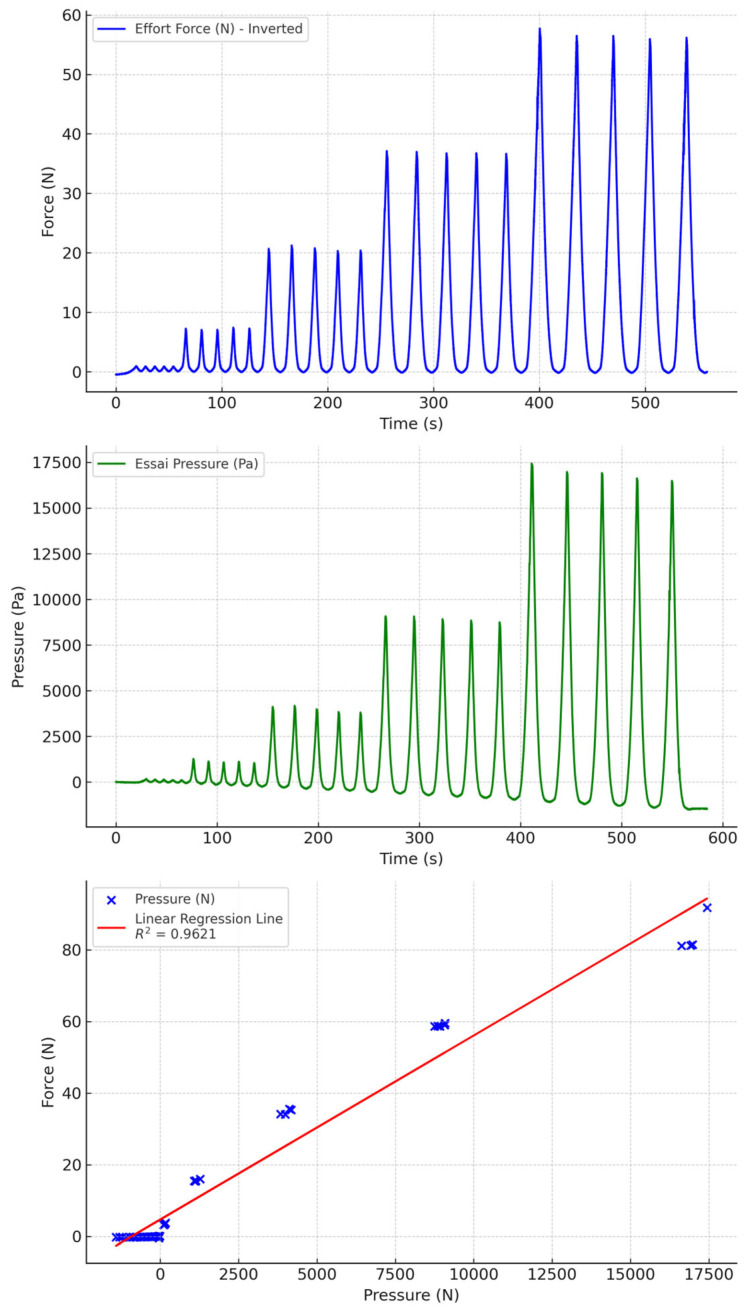
Water-filled PU tubing with non-biocompatible socks.

Water-filled PU tubing, whether combined with biocompatible or non-biocompatible silicone socks, results in less drift than air-filled tubing, but the results are still significant (Figure 8 and Figure 9). This drift is greater with biocompatible socks than with non-biocompatible socks.

### 3.3. Test with Water and PVC Tubing

**Figure 10:**
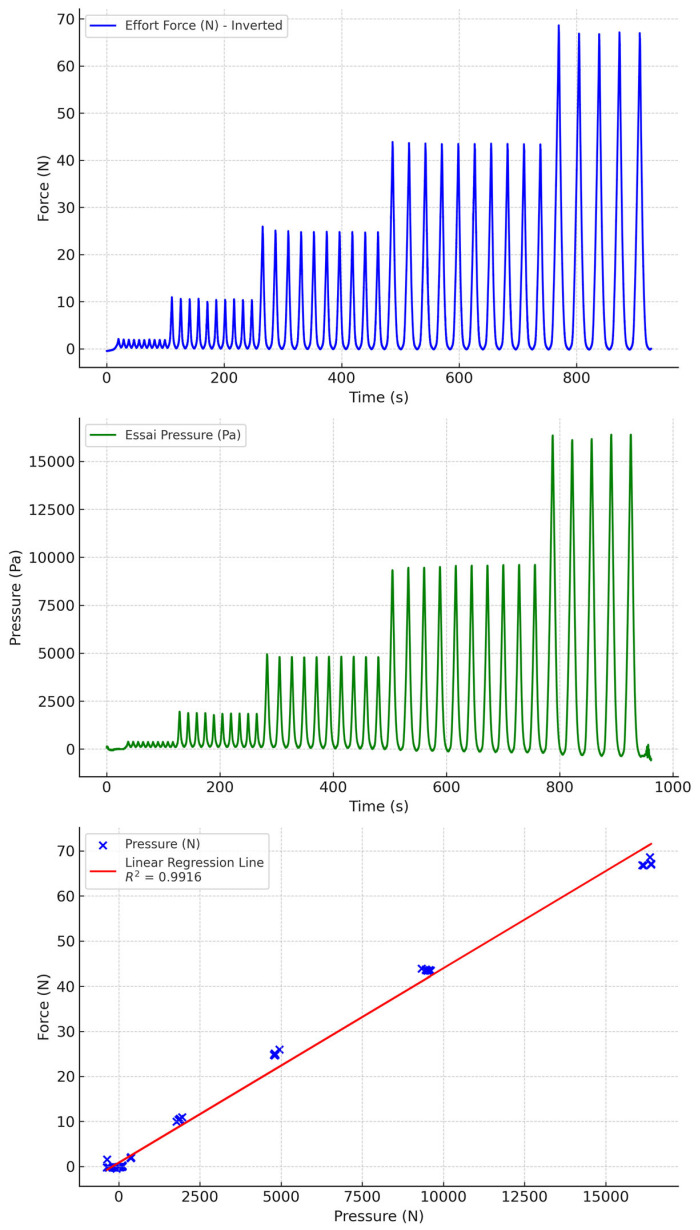
Water-filled PVC tubing with biocompatible socks.

**Figure 11:**
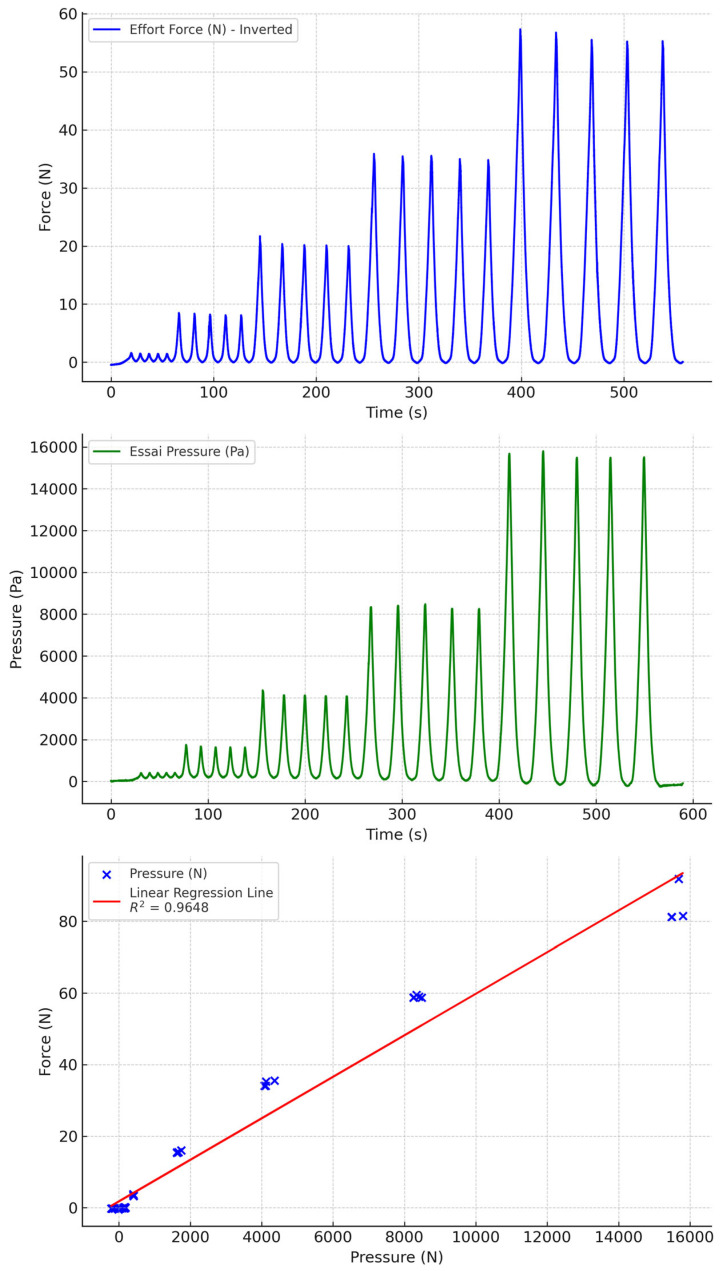
Water-filled PVC tubing with non-biocompatible socks.

Water-filled PVC tubing gave the best results (Figure 10 and Figure 11). Drift was minimal over the range of pressures measured. Moreover, the correlation between pressures and measured forces was very good. We also observed that biocompatible and non-biocompatible silicones gave similar measurements, with a slightly greater drift with biocompatible socks.

### 3.4. Test Summary

Table 1 summarizes all of the results obtained during the six bench tests. The combinations of materials and fluids that met our specifications (i.e., with a bias of less than 1% and 95% confidence interval limits of less than 10%) were water-filled PVC tubing with a biocompatible silicone sock, water-filled PVC tubing with a non-biocompatible silicone sock, and water-filled PU tubing with a non-biocompatible silicone sock.

## 4. Discussion

### 4.1. Advantages and Comparison to Analogues

To our knowledge, there is currently no existing system for measuring the pressure exerted by forceps blades on the fetal head. The latest estimates on fetal head compression date back to studies from 1959 to 1966 [20,21,22], which focused only on forces at the branches of the forceps rather than the direct contact surface between the blades and the fetal head. Our study offers a significant advancement by developing a device that measures the pressure over the entire contact surface of the forceps in real time, which has applications for both training and clinical practice. This novel approach allows for objective feedback and has the potential to improve the safety and efficacy of forceps delivery.

Our technique allows us to measure pressure across the entire contact surface of the forceps on the fetal head without gaps, albeit indirectly. We observed that, for a given contact surface and force, the pressure measured by our sensor (corresponding to the tubing pressure) varies based on the materials and fluids used. Nevertheless, our tests demonstrated high reliability and repeatability. When tested on a bench with a constant contact surface, we obtained a strong correlation between the force measured by a validated sensor and the pressure recorded by our device. The most effective configurations included water-filled PVC tubing with both biocompatible and non-biocompatible silicone support socks.

The purpose of this work was to create an adaptable pressure measurement device for use with existing clinical forceps. In training, this allows for objective analysis of the pressure applied on a fetal mannequin, highlighting areas for improvement in students’ initial training. The data collected will support external evaluation by both instructors and self-assessment, comparing each forceps delivery with a reference gesture developed by expert consensus. This provides a path to an improvement curve for each student. We are currently conducting mannequin tests with practitioners of varying experience levels to validate the device’s use in training simulations.

### 4.2. Limitations

This preliminary study focused on developing and validating a prototype in a controlled bench environment. While the device demonstrated reliable measurements in these tests, further validation is necessary in realistic settings. We are currently testing the device on mannequins with practitioners to evaluate its behavior in simulations, but no in vivo tests or clinical trials have been conducted yet. Additionally, while we used a range of materials, long-term durability, biocompatibility, and sterilization processes remain areas to be addressed.

### 4.3. Future Development and Research Directions

Our next steps include comprehensive mannequin tests to validate the device’s accuracy in simulated deliveries, paving the way for potential in vivo studies. We see this device as an invaluable training tool, offering objective feedback and tracking, which can enhance the skills of obstetrics trainees. In clinical practice, the device may provide real-time feedback, helping obstetricians optimize their technique during forceps-assisted deliveries. Ultimately, this system could reduce maternal and neonatal complications associated with forceps use and support safer delegation to less-experienced practitioners, allowing senior clinicians to monitor and assess procedures in real time.

## Figures and Tables

**Table 1:** Summary table of biases and upper and lower limits of 95% confidence intervals according to material and fluid used.

Material	R^2^	Bias	Lower Limit	Upper Limit
Water, PVC, biocompatible	0.9916	−0.0376	−3.5464	3.47121
Air, PVC, biocompatible	0.9303	0.0015	−14.3414	14.34435
Water, PVC, other	0.9648	−0.0082	−10.2217	10.20529
Water, PU, biocompatible	0.9501	0.0146	−12.2064	12.23564
Air, PU, biocompatible	0.9083	0.0003	−16.4485	16.44918
Water, PU, other	0.9621	−0.0973	−10.6924	10.49772

## Data Availability

Data are contained within the article.
